# Data sources and methods used to determine pretest probabilities in a cohort of Cochrane diagnostic test accuracy reviews

**DOI:** 10.1186/s12874-020-00952-w

**Published:** 2020-04-16

**Authors:** Michiel S. Oerbekke, Kevin Jenniskens, Rob J. P. M. Scholten, Lotty Hooft

**Affiliations:** 1 Knowledge Institute of the Federation of Medical Specialists, Utrecht, The Netherlands; 2 Cochrane Netherlands, Julius Center for Health Sciences and Primary Care, University Medical Center Utrecht, Utrecht University, Utrecht, The Netherlands; 3 Julius Center for Health Sciences and Primary Care, University Medical Center Utrecht, Utrecht University, Utrecht, The Netherlands

**Keywords:** Diagnostic test accuracy, Pretest probability, Pretest risk, Background prevalence, Review literature as topic, Absolute numbers, Normalized frequencies, Natural frequencies

## Abstract

**Background:**

A pretest probability must be selected to calculate data to help clinicians, guideline boards and policy makers interpret diagnostic accuracy parameters. When multiple analyses for the same target condition are compared, identical pretest probabilities might be selected to facilitate the comparison. Some pretest probabilities may lead to exaggerations of the patient harms or benefits, and guidance on how and why to select a specific pretest probability is minimally described. Therefore, the aim of this study was to assess the data sources and methods used in Cochrane diagnostic test accuracy (DTA) reviews for determining pretest probabilities to facilitate the interpretation of DTA parameters. A secondary aim was to assess the use of identical pretest probabilities to compare multiple meta-analyses within the same target condition.

**Methods:**

Cochrane DTA reviews presenting at least one meta-analytic estimate of the sensitivity and/or specificity as a primary analysis published between 2008 and January 2018 were included. Study selection and data extraction were performed by one author and checked by other authors. Observed data sources (e.g. studies in the review, or external sources) and methods to select pretest probabilities (e.g. median) were categorized.

**Results:**

Fifty-nine DTA reviews were included, comprising of 308 meta-analyses. A pretest probability was used in 148 analyses. Authors used included studies in the DTA review, external sources, and author consensus as data sources for the pretest probability. Measures of central tendency with or without a measure of dispersion were used to determine the pretest probabilities, with the median most commonly used. Thirty-two target conditions had at least one identical pretest probability for all of the meta-analyses within their target condition. About half of the used identical pretest probabilities were inside the prevalence ranges from all analyses within a target condition.

**Conclusions:**

Multiple sources and methods were used to determine (identical) pretest probabilities in Cochrane DTA reviews. Indirectness and severity of downstream consequences may influence the acceptability of the certainty in calculated data with pretest probabilities. Consider: whether to present normalized frequencies, the influence of pretest probabilities on normalized frequencies, and whether to use identical pretest probabilities for meta-analyses in a target condition.

## Background

Diagnostic tests are essential to clinicians in their daily practice. Test results inform about the preferred healthcare pathway to, ideally, cure a patient from disease. The optimal way to understand a diagnostic test’s performance and the downstream consequences for patients is through a test-treatment randomized controlled trial. Such trials provide comparative information on health outcomes (both harms and benefits) of healthcare pathways initiated by the outcome of the diagnostic tests or strategies. However, test-treatment randomized controlled trials are methodologically complex [1]. Diagnostic test accuracy (DTA) studies are usually an alternative to these complex trials and can be summarized in systematic reviews. DTA reviews include primary cross-sectional studies using the diagnostic test of interest and aggregate data by meta-analysis so that a pooled sensitivity and specificity is presented. Sensitivity is the proportion of persons with the target condition that are correctly classified by the index test, while specificity is the proportion of correctly classified persons without the target condition. Persons who are false negatively misclassified might not receive an intervention when they should have. Further diagnostic testing may be indicated which is possibly more burdensome or more harmful (e.g. the next diagnostic test is more invasive or may involve nuclear imaging). Persons who were false positively misclassified are falsely diagnosed with the presence of the target condition. Then, the provided intervention may be unnecessary and potentially burdensome, depending on the nature of the intervention. Complications from the intervention may arise and complaints may persist, potentially resulting in a late diagnosis of the actual present target condition. Further treatment for the actual target condition may then have more risks or complications compared to early diagnosis and intervention.

From literature it seems that clinicians have trouble interpreting accuracy parameters such as sensitivity and specificity [2]. To facilitate the interpretation of DTA results absolute numbers of true/false positives and true/false negatives can be presented in a hypothetical cohort of e.g. 1000 persons, which is also known as normalized frequencies [2]. However, to calculate normalized frequencies a pretest probability (i.e. the disease prevalence in the hypothetical cohort) needs to be determined. The normalized frequencies are then calculated and reported, whereafter the diagnostic test’s end-user can interpret whether the test performance is acceptable in terms of true or false positives and negatives. Such normalized frequencies are usually presented in summary of findings tables in Cochrane DTA Reviews and in the evidence tables from the Grading of Recommendations Assessment, Development and Evaluation (GRADE) [3].

These normalized frequencies are not only important for clinicians, but also for guideline boards and policy makers. Decisions whether or not to recommend the use of a diagnostic test in a guideline or decisions about health care restitution may be influenced by the presented normalized frequencies. However, normalized frequencies are dependent on the chosen pretest probability. Using different pretest probabilities while the sensitivity and specificity remain constant may lead to an exaggeration of the estimated patient harms and/or benefits due to varying absolute numbers of (mis) classifications as calculated in the hypothetical cohort. Furthermore, the normalized frequencies from a hypothetical cohort can be more directly compared when using identical pretest probabilities for multiple tests within a target condition. However, selecting an identical pretest probability for multiple meta-analyses in a single target condition could be challenging. The selected pretest probability might not lie in the prevalence range of every meta-analysis and, therefore, extrapolation of data may occur, potentially decreasing the certainty of the presented normalized frequencies.

While GRADE does not suggest a specific method to determine a pretest probability in its handbook [3, 4] the Cochrane Handbook does propose some methods (e.g. the median disease prevalence or the prevalence from disease registries) although a rationale to use a specific method is not given [5]. Because guidance in determining a pretest probability is minimally described, it is unknown what data sources (i.e. the data on which a pretest probability is based) and methods are actually used to determine a pretest probability in DTA reviews. Therefore, the aim of this study was to assess the data sources and methods used in a cohort of Cochrane DTA reviews to determine pretest probabilities for the facilitation of pooled DTA accuracy parameter interpretation and to provide some considerations for the use of normalized frequencies. A secondary aim was to assess the use of identical pretest probabilities in multiple analyses within the same target condition, necessary for the comparison of test performances.

## Methods

### Cohort definition

The Cochrane Database of Systematic Reviews (CDSR) was accessed through the Cochrane Library. The Cochrane Collaboration has pioneered methods for DTA reviews and the CDSR contains DTA reviews since 2008 [6, 7]. Cochrane DTA reviews published in the period from 2008 up to and including January 2018 were potentially eligible to enter the cohort. To obtain DTA reviews the CDSR was browsed by the topic ‘Diagnosis’, while protocols and intervention or methodology reviews were excluded through limiters in the search engine interface. A DTA review was included in the cohort when it reported at least one meta-analytic estimate of sensitivity and/or specificity (i.e. either retrieved with a bivariate model or by using a hierarchical summary receiver operating characteristic model) as a primary analysis in the presented tables, which were usually summary of findings tables. The screening and selection of eligible DTA reviews was performed by one author (MSO) and checked by the other authors (KJ, RJPMS, LH).

### Data extraction

The method for determining the pretest probability itself was recorded. For example, this could be the use of the mean or median disease prevalence (from studies included for a target condition). General characteristics (e.g. title, publication year), the number of meta-analyses in the review, whether a pretest probability was used, the number of pretest probabilities used (if applicable), the source of data for determining the pretest probability, and the method used for selecting pretest probabilities (if applicable) were extracted by one author (MSO) and checked by the other authors (KJ, RJPMS, LH). We only extracted data sources and methods for pretest probabilities from primary meta-analyses (usually presented in summary of findings tables). Meta-analyses with the purpose of being sensitivity analyses when excluding certain studies or with the purpose of heterogeneity analysis were not considered for data-extraction, even when they were presented in the summary of findings table. When a disease prevalence was reported but not used to interpret the sensitivity and/or specificity in some manner, the disease prevalence was not considered as a pretest probability. Descriptive statistics were performed in IBM SPSS Statistics for Windows (Version 21, 2012, Armonk, NY: IBM Corp.).

### Categorization of data sources

The first step in the categorization of extracted data was to divide the included meta-analyses into two groups; One group of analyses where no pretest probability was used and one group of analyses where a pretest probability was used. Next, for every meta-analysis that used a pretest probability the source of the pretest probability was determined. The pretest probability could be determined based on the studies that were included in the review, from external sources, or from author consensus. The data source of a pretest probability was categorized as unclear when the source could not be determined. Further categorization of data sources took place when the pretest probability was determined from included studies. Pretest probabilities used to interpret accuracy meta-analyses could then be determined from all studies included for the target condition, from all studies used per test/analysis for a target condition, from all included studies, from all included studies and from an unclear source, from all included studies and from studies with a low risk of bias, or only from included studies that reported the disease prevalence. Further categorization also took place when the pretest probability was determined from external sources. Pretest probabilities could then be determined from published scientific literature, from the World Health Organization’s suggestions, or from a guideline. The data source of pretest probabilities was categorized as ‘author consensus’ when a pretest probability was assumed by de review’s authors and not based on included studies or external sources. See Additional File 2 for a description and example of each category. Methods to determine a pretest probability from the observed data sources were recorded and counted. Methods in external sources were not recorded, since these methods were not used in the Cochrane DTA reviews but in the external source.

### Identical pretest probabilities within a target condition

Target conditions in DTA reviews that had multiple meta-analyses for the same target condition were identified to observe the use of identical pretest probabilities.

One author (MSO) extracted the following data from the multiple meta-analyses within a target condition: whether identical pretest probabilities were used for all of the meta-analyses within a target condition, and whether the prevalence ranges of all of the individual meta-analyses for a target condition contained the selected identical pretest probability or not. When the prevalence range of individual meta-analyses in a single target condition did not contain the selected identical pretest probability, a justification by the review authors was sought in the review. Data extraction was checked by a second author (KJ). Prevalence ranges in individual meta-analyses were calculated from data in the DTA review’s appendices when not reported in the text.

## Results

### Cohort description

**Fig. 1:**
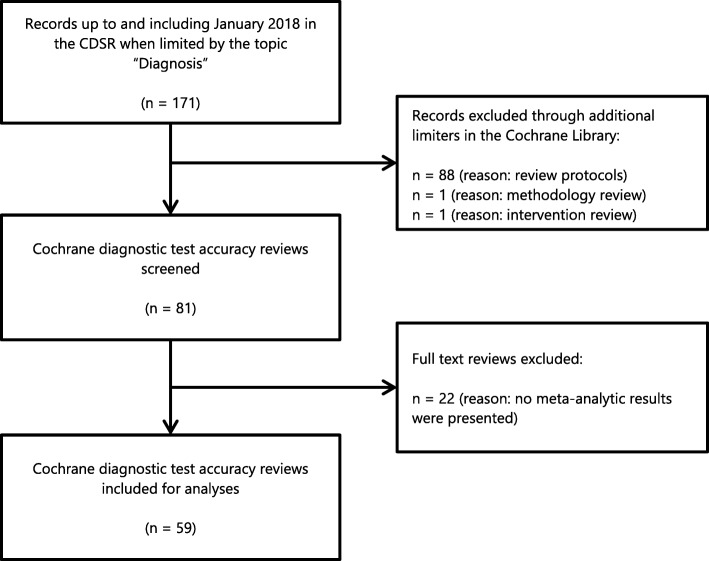
Cohort formation. Flow diagram showing the formation of the cohort and reasons for exclusion. CDSR: Cochrane Database of Systematic Reviews

The CDSR contained 171 documents on the topic ‘Diagnosis’. There were 81 reviews left after excluding 88 review protocols and 2 reviews on interventions and methodology. After screening the full text an additional 22 DTA reviews were excluded as no meta-analytic results were presented (referenced in Additional File 1). Consequently, 59 Cochrane DTA reviews were included in the cohort (Fig. 1 and Additional File 1). The 59 DTA reviews in the cohort contained 308 meta-analyses (see Table 1). The number of meta-analyses ranged from 1 to 34 (median: 3) per review. In 16 reviews (16/59, 27.1%) there were 150 meta-analyses (150/308, 48.7%) that did not use a pretest probability. Thirty-nine reviews (39/59, 66.1%) had 143 meta-analyses (143/308, 46.4%) where a pretest probability was used. Four reviews (4/59, 6.8%) contained 15 meta-analyses for which a pretest probability was used in 5 analyses. Therefore, a total of 160 analyses (160/308, 51.9%) were found where no pretest probability was used and 148 analyses (148/308, 48.1%) were found where at least one pretest probability was used.

**Table 1:** General characteristics

	Reviews	Meta-analyses	Number of meta-analyses per DTA review	Number of pretest probabilities used per meta-analysis^a^
	n	n (% using a pretest probability)	Median (range)	Median (range)
**Cochrane DTA reviews**				
Total included DTA reviews	59	308 (48.1)	3 (1–34)	1 (1–6)
Reviews not using a pretest probability at all	16	150 (0)	4 (2–34)	-^a^
Reviews using a pretest probability for all pooled analyses	39	143 (100)	3 (1–16)	1 (1–5)
Reviews reporting analyses with and without pretest probabilities	4	15 (33.3)	3.5 (3–5)	3 (1–6)

*DTA* Diagnostic Test Accuracy, *IQR* Interquartile Range

^a^Could not be calculated since there were no analyses using a pretest probability

### Sources of pretest probabilities

**Fig. 2:**
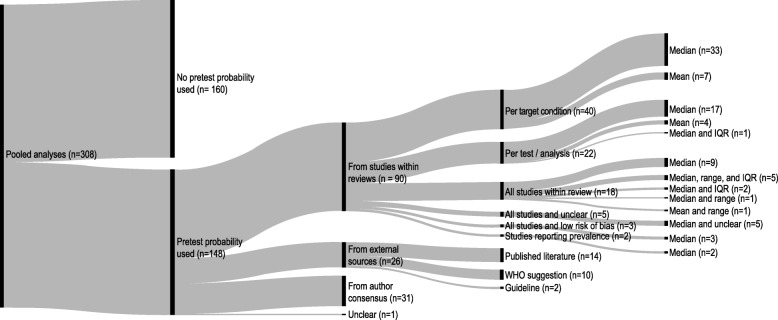
Data sources and methods for determining pretest probabilities in Cochrane Diagnostic Test Accuracy reviews. Sankey plot showing which data sources and methods were used to determine the pretest probability in Cochrane Diagnostic Test Accuracy reviews. IQR: Interquartile Range, WHO: World Health Organization

In the 148 analyses in which a pretest probability was used three main categories of data sources were distinguished (Fig. 2). In 90 (60.8%) of the 148 analyses the pretest probability was determined from included studies, in 26 analyses (26/148, 17.6%) from external sources, and from author consensus in 31 analyses (31/148, 20.9%). In one analysis the data source was unclear. When the included studies in the review were used to determine one or multiple pretest probabilities, the data source could be further differentiated. A pretest probability was determined from all studies included for a target condition in 40 analyses (40/90, 44.4%), from studies used per test/analysis for a target condition in 22 analyses (22/90, 24.4%), or from all studies in the systematic review in 18 analyses (18/90, 20%). Five other analyses (5/90, 5.6%) had multiple pretest probabilities where some were determined from all studies in the systematic reviews and some from an unclear source. Three analyses (3/90, 3.3%) had multiple pretest probabilities, where some were obtained from all studies in the systematic review and some from the included low risk of bias studies. A pretest probability was determined in 2 analyses (2/90, 2.2%) from the studies that reported their prevalence. When the pretest probability was obtained from external sources, the data sources could also be further differentiated. In 14 analyses (14/26, 53.8%) the pretest probability was a disease prevalence reported in published scientific literature, in 10 analyses (10/26, 38.5%) a suggestion by the WHO, and in 2 analyses (2/26, 7.7%) a disease prevalence reported in a guideline.

### Methods for determining a pretest probability

Pretest probabilities based on studies within the review were determined by using measures of central tendency (e.g. median) whether or not combined with measures of dispersion (e.g. range). Using multiple methods resulted in multiple pretest probabilities for a single analysis (e.g. using a median with a range results in three pretest probabilities). The median was used individually in 64 analyses (64/90, 71.1%) or together with the interquartile range in 3 analyses (3/90, 3.3%), with the range in 1 analysis (1/90, 1.1%), with the range and interquartile range in 5 analyses (5/90, 5.6%), or together with other estimates of pretest probabilities for which the method was unclear in 5 analyses (5/90, 5.6%). The mean was used individually in 7 analyses (7/90, 7.8%) or together with the range in 1 analysis (1/90, 1.1%). Fig. 2 shows the methods per data source and the number of analyses in where these methods were used.

### Identical pretest probabilities for multiple meta-analyses within target conditions

**Fig. 3:**
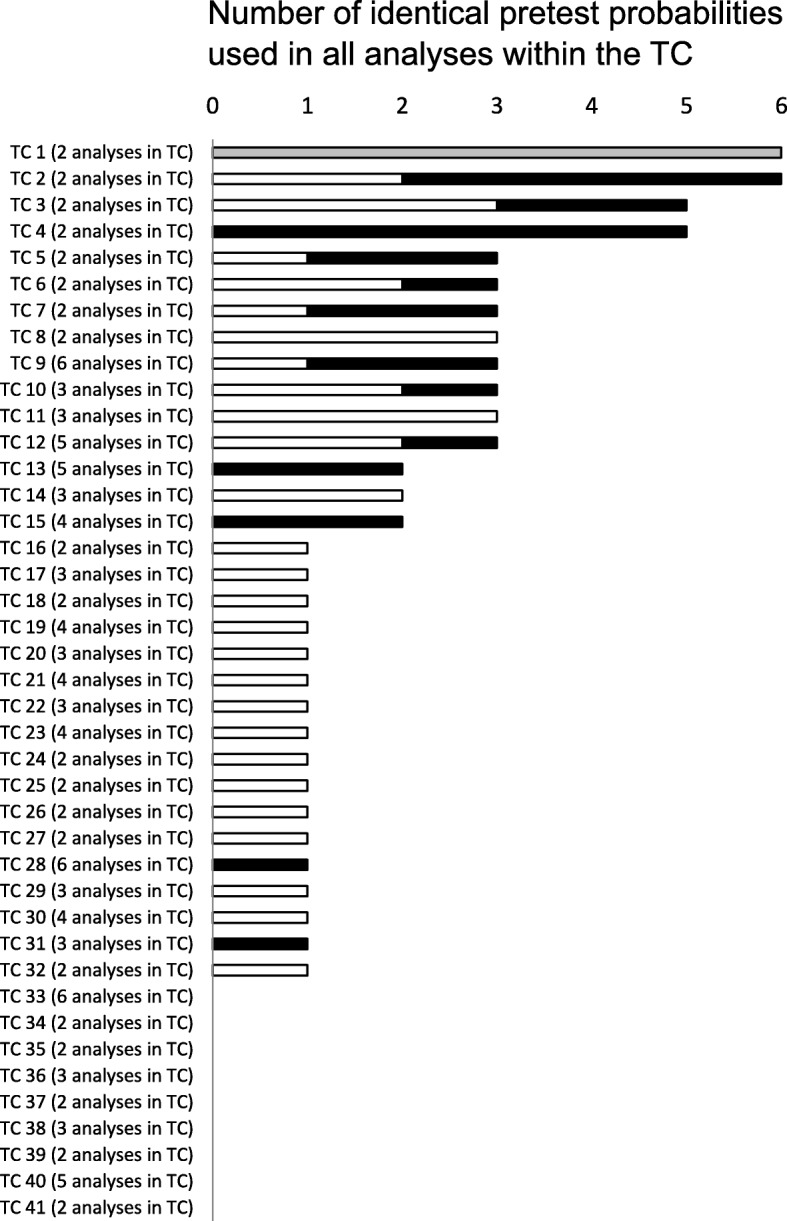
Identical pretest probabilities within target conditions. The figure shows the number of target conditions with two or more analyses and the use of identical pretest probabilities for all of the meta-analyses within a target condition. Black bars indicate the number of pretest probabilities that were outside the prevalence range of at least one meta-analysis for the target condition. White bars indicate the number of pretest probabilities that were inside the prevalence ranges of all individual meta-analyses for that specific target condition. The gray bar indicates that it was unclear whether these identical pretest probabilities were inside or outside the disease prevalence ranges. No bars were drawn in the figure when there were no identical pretest probabilities used for all of the meta-analyses within a target condition. DTA: Diagnostic Test Accuracy, TC: Target Condition

There were 41 target conditions having two or more analyses which might have used identical pretest probabilities to compare test performances for tests in the same diagnostic role within a target condition (Fig. 3). Nine target conditions (9/41, 22%) did not have identical pretest probabilities in their analyses. One target condition (1/41, 2.4%) had six identical pretest probabilities for its two meta-analyses, however it was unclear whether the prevalence ranges of the two meta-analyses contained the pretest probability estimates. Thirty-two target conditions (32/41, 78%) had at least one identical pretest probability (range: 1–6) that was used for all of the meta-analyses within their respective target condition. Sixty-nine identical pretest probabilities within target conditions were identified. Thirty-seven pretest probabilities (37/69, 53.6%) fell in the prevalence range of all of the individual meta-analyses within that target condition. However, 26 pretest probabilities (26/69, 37.7%) in 11 DTA reviews fell outside the prevalence range from at least one meta-analysis within that target condition. In 5 of these 11 DTA reviews pretest probabilities were used to show test performances in different scenarios (i.e. referral scenarios, low/high risk scenarios, newly/previously treated cases, geographical locations, adults/children).

## Discussion

### Summary of key findings

A total of 59 Cochrane DTA reviews were included to assess the data sources and methods used to determine pretest probabilities and to assess the use of identical pretest probabilities in multiple analyses within a target condition. Various sources and methods to determine a pretest probability were found. Sixteen DTA reviews did not use a single pretest probability. Almost half of the observed meta-analyses used at least one pretest probability (range: 1–6 pretest probabilities) to facilitate the interpretability of the results. The median was the most used method to determine a pretest probability. Thirty-nine target conditions contained two or more analyses and used at least one identical pretest probability for all of its analyses (range: 1–6 pretest probabilities). Twenty-six of the identical pretest probabilities (37.7%) fell outside the disease prevalence range of at least one analysis within the target condition.

### Interpretation of results

The Cochrane Handbook for Systematic Reviews of Diagnostic Test Accuracy proposes to use the median prevalence of the included studies or external sources as methods to select a pretest probability [5]. Indeed, the median was used more than any other method in Cochrane DTA reviews when the pretest probability was determined based on studied included in the review. External sources were also used in 26 analyses. The Cochrane Handbook also suggests the use of disease registries for selecting a representative pretest probability, but no such method was specifically mentioned in the included Cochrane DTA reviews. Observed prevalence from disease surveillance systems and epidemiological surveys by the WHO, however, might be interpreted as disease registries as well. The Cochrane Handbook states that a representative pretest probability may be derived from the included studies only when the studies are representative for the target setting [5], in which case the selected pretest probability will fall within the prevalence range of included studies. However, a pretest probability may be selected that falls outside the disease prevalence range from the included studies. This might also be the case when authors wish to use an identical pretest probability for several meta-analyses within the same target condition and use, for example, the median prevalence from all included studies for that target condition (e.g. the median of all included studies may fall outside some prevalence ranges when these ranges do not overlap sufficiently). A pretest probability could be representative for the target setting, but it might not necessarily be an appropriate pretest probability in the context of the disease prevalence range from the included studies in a meta-analysis. When a representative pretest probability falls outside the disease prevalence range it might be considered indirect, since the observed data did not contain that pretest probability. When considering the observed data, a more data-appropriate pretest probability from within the disease prevalence range from the meta-analysis itself might be selected. Therefore, a representative and a data-appropriate pretest probability are not necessarily the same (see Example 1 in Additional File 3). Appropriateness for the data in this case means to not extrapolate outside of the data in the meta-analysis, because there is uncertainty about the test’s performance outside the observed data.

### Study limitations

A potential limitation of this study is that only Cochrane DTA reviews were included. Since the Cochrane Handbook proposes to use the median it was beforehand likely to observe that the median is being preferred in Cochrane DTA reviews. Different methods and data sources for determining pretest probabilities in non-Cochrane DTA reviews could have been missed. Furthermore, DTA review authors may also choose to aid the interpretation of non-meta-analytic results. For example, normalized frequencies may also have been calculated for single DTA studies in the review. However, only the use of pretest probabilities in meta-analyses was assessed in the current study. The potential issues regarding the data sources and methods for choosing a pretest probability remain the same for single studies as for meta-analytic estimates. Data sources and methods for determining pretest probabilities in the absence of a meta-analysis are therefore still unknown. However, when there were data sources or methods that this study did not address, it adds to the impression that there is no consensus on what data source and method to use for determining a representative or appropriate pretest probability.

### Implications for using pretest probabilities

From the results of this study no clear guidance can be given on what source or what method should be used for determining a pretest probability. Furthermore, it is unknown if a pretest probability outside the disease prevalence range is problematic in clinical reality. Whether or not it is problematic may also be context dependent, as for some target conditions a certain number of misclassifications are more acceptable than for target conditions where misclassifications have severe downstream consequences. Even if it turns out to be clinically problematic in future research, it presently might still be best practice to facilitate the interpretability of diagnostic accuracy parameters by presenting normalized frequencies. Furthermore, there are some considerations which may be taken in to account when presenting results in DTA reviews.

First to consider is whether to provide a way for end-users to interpret the presented accuracy parameters, as it was observed in this study that about half of all meta-analyses were not accompanied with normalized frequencies from a hypothetical cohort. Literature shows that interpreting diagnostic test accuracy parameters may be troublesome for its users and therefore normalized frequencies may be useful [2]. However, choosing pretest probabilities to calculate normalized frequencies is not without difficulties and therefore it is uncertain whether normalized frequencies are trustworthy enough for all decision-making (see the second consideration). The need for interpretability versus the certainty of and need for a truthful representation might determine whether normalized frequencies are calculated. We might accept more or less certainty in the normalized frequencies depending on their degree of indirectness and the severity of downstream consequences for misclassifications (see Example 2 in Additional File 3). However, not facilitating the interpretation of accuracy parameters also complicates the judgement by clinicians, policy makers, and guideline boards whether to use the diagnostic test.

Secondly, consider giving thought about the influence of the method of selecting the pretest probability on the normalized frequencies from the hypothetical cohort. A guideline board may base their decision about whether or not to recommend a test for clinical practice on the presented normalized frequencies. It is important to understand that different pretest probabilities will result in different normalized frequencies while the sensitivity and specificity remain constant (see Example 3 in Additional File 3), potentially influencing the decision-making in practice, policy or guidelines.

Thirdly, when there are multiple meta-analyses for the same target condition, consider whether to use an identical pretest probability in each of those analyses so that the normalized frequencies can be compared. Ideally the selected pretest probabilities fall inside all of the disease prevalence ranges from all individual meta-analyses within the target condition, although this might not be feasible for every scenario (e.g. when the disease prevalence ranges from the meta-analyses do not overlap). However, from this study no guidance can be provided on whether an identical pretest probability is suitable for all of the disease prevalence ranges in the analyses, even when the pretest probability falls inside all prevalence ranges.

Providing clinicians, policy makers, and guideline boards with methods to facilitate the interpretation of DTA results is not only important for them, but ultimately also for patients who undergo diagnostic tests. Different pretest probabilities will result in different normalized frequencies. However, it is not known whether differences in normalized frequencies caused by the use of different pretest probabilities actually impacts decision-making and whether it will then clinically harm of benefit patients. The future direction of research in this area could focus on whether different pretest probabilities will actually result in a different clinical decision, guideline recommendation, or policy change. Furthermore, future research could focus on developing other strategies for accuracy parameters so that they are both interpretable and helpful when research shows that calculating normalized frequencies may not be beneficial for actual decision-making by clinicians, policy makers, or guideline boards.

## Conclusions

Various data sources and methods are used to obtain pretest probabilities, without consensus on which data source or method to use. Identical pretest probabilities were used in some DTA reviews, where test performances for a target condition could be directly compared in about half of the identical pretest probabilities. The certainty of presented data calculated with pretest probabilities is influenced by indirectness. Indirectness is probably more acceptable in situations where there are less severe downstream consequences, but the reduction of certainty should be acknowledged. There are three considerations that might be taken in to account when presenting DTA results: consider whether or not to present normalized frequencies from a hypothetical cohort; consider the influence of the chosen method for selecting a pretest probability on the normalized frequencies on which a clinical decision, guideline recommendation or policy change may be based, and consider to use identical pretest probabilities that fall within the range of the selected studies when there are multiple meta-analyses for the same target condition.

## Supplementary information

**Additional file1.** Included and excluded reviews. An Excel table showing the included and excluded Cochrane DTA reviews with exclusion reasons and URLs linking to the reviews in the Cochrane Database of Systematic Reviews.

**Additional file2.** Examples of data source categories. A short description of the observed categories and an example in each category.

**Additional file3.** Examples based on real-world data from a Cochrane DTA review of the changes in normalized frequencies when using different pretest probabilities.

## Data Availability

The datasets used and/or analyzed during the current study are available from the corresponding author on reasonable request.

## Abbreviations

CDSR: Cochrane database of systematic reviews
DTA: Diagnostic test accuracy
GRADE: Grading of recommendations, assessment, development and evaluation
WHO: World health organization
