# Supplementary material for: Data sources and methods used to determine pretest probabilities in a cohort of Cochrane diagnostic test accuracy reviews
Source: BMC Med Res Methodol. 2020 Apr 16;20:85. doi: 10.1186/s12874-020-00952-w (PMC7161259; doi:10.1186/s12874-020-00952-w)
Supplement: Supplementary file 2 — Additional file2. Examples of data source categories. A short description of the observed categories and an example in each category. [file 12874_2020_952_MOESM2_ESM.docx]

**Additional file 2 – Examples of data source categories**

**From included studies**

*From all studies included for the target condition*

There are multiple target conditions in the systematic review and a target condition has one or multiple index tests. All studies from all analyses for a single target condition were used to determine a pretest probability. See Lenza et al. (2013) for an example [1].

*From studies used per test/analysis for a target condition*

A target condition has multiple index tests. For each analysis of the index test the included studies for that index test were used to determine a pretest probability. See Colli et al. (2017) for an example [2].

*From all studies in the systematic review across all target conditions*

A review used all of the included studies for analyses across all of the target conditions to determine a pretest probability. Analyses were also placed in this category if there was only one target condition defined in the systematic review and a pretest probability was determined from all included studies for that target condition, unless specifically stated otherwise by the authors. See Leeflang et al. (2015) for an example [3].

*From all studies in the systematic review and from an unclear source*

An analysis used two pretest probabilities to calculate normalized frequencies. One of the pretest probabilities was determined from all of the included studies for analyses across all target conditions, while the other pretest probability had an unclear data source. See Abba et al. (2011) for an example [4].

*From all studies in the systematic review and only from studies with a low risk of bias*

An analysis used two pretest probabilities to calculate normalized frequencies. One pretest probability was determined by all of the included studies for analyses across all target conditions, while the other pretest probability was calculated solely from studies with a low risk of bias. See Colli at al. (2014) for an example [5].

*Only from included studies that reported the disease prevalence*

Only studies that reported their sample’s disease prevalence were used to determine the pretest probability. See Ritchie et al. (2017) for an example [6].

**From external sources**

*From published scientific literature*

The pretest probability used in the systematic review was based on or informed by the disease prevalence as reported in published scientific literature. See Wijedoru et al. (2017) for an example [7].

*From a WHO suggestion*

The pretest probability used in the systematic review was based on or informed by the disease prevalence as suggested by the WHO. See Steingart et al. (2014) for an example [8].

*From a guideline*

The pretest probability used in the systematic review was based on or informed by the disease prevalence as reported in a guideline. See Wang et al. (2011) for an example [9].

**Author consensus**

It was considered author consensus when authors determined a pretest probability based on an assumption while not calculated from included studies or taken from external sources (including suggestions from external parties). See Shaikj et al. (2016) for an example [10].

**REFERENCES**

1. Lenza M, Buchbinder R, Takwoingi Y, Johnston RV, Hanchard NC, Faloppa F. Magnetic resonance imaging, magnetic resonance arthrography and ultrasonography for assessing rotator cuff tears in people with shoulder pain for whom surgery is being considered. *The Cochrane database of systematic reviews* 2013(9):Cd009020. doi: 10.1002/14651858.CD009020.pub2 [published Online First: 2013/09/26]

2. Colli A, Gana JC, Yap J, Adams-Webber T, Rashkovan N, Ling SC, Casazza G. Platelet count, spleen length, and platelet count-to-spleen length ratio for the diagnosis of oesophageal varices in people with chronic liver disease or portal vein thrombosis. *The Cochrane database of systematic reviews* 2017;4:Cd008759. doi: 10.1002/14651858.CD008759.pub2 [published Online First: 2017/04/27]

3. Leeflang MM, Debets-Ossenkopp YJ, Wang J, Visser CE, Scholten RJ, Hooft L, Bijlmer HA, Reitsma JB, Zhang M, Bossuyt PM, Vandenbroucke-Grauls CM. Galactomannan detection for invasive aspergillosis in immunocompromised patients. *The Cochrane database of systematic reviews* 2015(12):Cd007394. doi: 10.1002/14651858.CD007394.pub2 [published Online First: 2015/12/31]

4. Abba K, Deeks JJ, Olliaro P, Naing CM, Jackson SM, Takwoingi Y, Donegan S, Garner P. Rapid diagnostic tests for diagnosing uncomplicated P. falciparum malaria in endemic countries. *The Cochrane database of systematic reviews* 2011(7):Cd008122. doi: 10.1002/14651858.CD008122.pub2 [published Online First: 2011/07/08]

5. Colli A, Gana JC, Turner D, Yap J, Adams-Webber T, Ling SC, Casazza G. Capsule endoscopy for the diagnosis of oesophageal varices in people with chronic liver disease or portal vein thrombosis. *The Cochrane database of systematic reviews* 2014(10):Cd008760. doi: 10.1002/14651858.CD008760.pub2 [published Online First: 2014/10/02]

6. Ritchie C, Smailagic N, Noel-Storr AH, Ukoumunne O, Ladds EC, Martin S. CSF tau and the CSF tau/ABeta ratio for the diagnosis of Alzheimer's disease dementia and other dementias in people with mild cognitive impairment (MCI). *The Cochrane database of systematic reviews* 2017;3:Cd010803. doi: 10.1002/14651858.CD010803.pub2 [published Online First: 2017/03/23]

7. Wijedoru L, Mallett S, Parry CM. Rapid diagnostic tests for typhoid and paratyphoid (enteric) fever. *The Cochrane database of systematic reviews* 2017;5:Cd008892. doi: 10.1002/14651858.CD008892.pub2 [published Online First: 2017/05/26]

8. Steingart KR, Schiller I, Horne DJ, Pai M, Boehme CC, Dendukuri N. Xpert(R) MTB/RIF assay for pulmonary tuberculosis and rifampicin resistance in adults. *The Cochrane database of systematic reviews* 2014(1):Cd009593. doi: 10.1002/14651858.CD009593.pub3 [published Online First: 2014/01/23]

9. Wang LW, Fahim MA, Hayen A, Mitchell RL, Baines L, Lord S, Craig JC, Webster AC. Cardiac testing for coronary artery disease in potential kidney transplant recipients. *The Cochrane database of systematic reviews* 2011(12):Cd008691. doi: 10.1002/14651858.CD008691.pub2 [published Online First: 2011/12/14]

10. Shaikh N, Spingarn RB, Hum SW. Dimercaptosuccinic acid scan or ultrasound in screening for vesicoureteral reflux among children with urinary tract infections. *The Cochrane database of systematic reviews* 2016;7:Cd010657. doi: 10.1002/14651858.CD010657.pub2 [published Online First: 2016/07/06]
