# Supplementary material for: Data sources and methods used to determine pretest probabilities in a cohort of Cochrane diagnostic test accuracy reviews
Source: BMC Med Res Methodol. 2020 Apr 16;20:85. doi: 10.1186/s12874-020-00952-w (PMC7161259; doi:10.1186/s12874-020-00952-w)
Supplement: Supplementary file 3 — Additional file3. Examples based on real-world data from a Cochrane DTA review of the changes in normalized frequencies when using different pretest probabilities. [file 12874_2020_952_MOESM3_ESM.docx]

**Additional file 3 – Examples**

**Example 1 – Representativeness for a setting and data-appropriateness**

DTA review authors may choose to use a pretest probability that is representative for the setting in which the diagnostic test is used. However, this representative pretest probability may fall outside the disease prevalence range from the observed data. External estimates of prevalence might be used when included studies do not match the population to which the test is to be applied in practice. The review authors then need to justify their choice and should acknowledge that there might be more uncertainty in their conclusion in relation to the downstream consequences of using the test in a hypothetical cohort. Although the pretest probability is representative for their target population (or setting), the meta-analysis of test performance measures includes studies that potentially do not closely match their situation. We know that patient spectrum affects prevalence, and therefore also performance measures as sensitivity and specificity. Using external pretest probabilities may lead to exaggeration or underestimation of the patient harms or benefits in the hypothetical cohort. Even when there is high confidence in the pooled accuracy estimates (e.g. assessed with GRADE), there might possibly be some indirectness in the calculations made with pretest probabilities outside the disease prevalence range.

For example, in the Cochrane DTA review by Wijedoru et al. (2017) there were 5 meta-analytic results presented in the summary of findings table for diagnostic tests in (para)typhoid fever. Three representative pretest probabilities were selected by the review authors for geographical regions and populations: 1% for children in Africa, 10% for adults and children in Africa, and 30% for Asia. The prevalence range for each meta-analysis is shown in Figure A1. From the figure we can see that the prevalence ranges of all meta-analyses contain the representative pretest probabilities of 10% and 30%. Both pretest probabilities are appropriate for the observed data as well, since the prevalence ranges of all the meta-analyses contain both 10% and 30%. For children in Africa the representative pretest probability is 1%. The prevalence ranges from three meta-analyses contain this representative pretest probability and are, therefore, appropriate for the data in these three analyses. The representative pretest probability of 1% is not appropriate in the context of the observed data in the Typhidot (subset 2) and Test-it analyses, since the disease prevalence ranges of both meta-analyses did not contain the representative pretest probability of 1%. Therefore, the normalized frequencies are calculated using the pooled accuracy estimates that are not based on data about the test’s performance at a prevalence of 1%. Nonetheless, the choice and justification to use the pretest probability of 1% is legitimate and it is unsure whether presenting normalized frequencies with pretest probabilities outside the prevalence range is actually harmful in clinical reality.

| 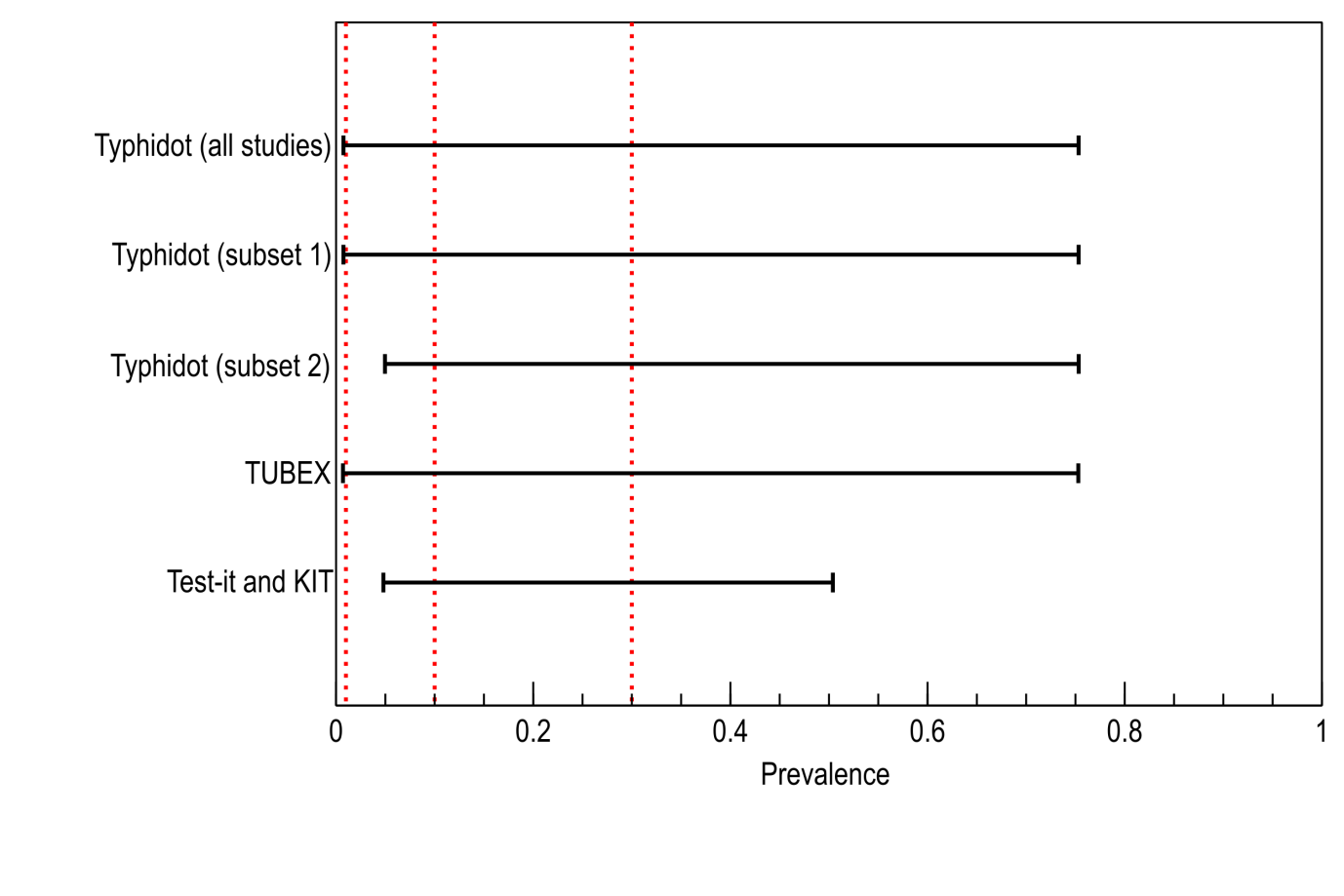**Figure A1 – Prevalence ranges of meta-analyses within a target condition.** Black lines represent the prevalence range; Red dotted lines represent the pretest probabilities at 1%, 10%, and 30% selected by the review authors. |
| --- |

**Example 2 – The need for interpretability versus the certainty of the data**

Several situations might lower the confidence in the certainty of calculated downstream consequences (effects) of test usage. However, the need for high certainty/confidence depends on the severity of the downstream consequences of misclassifications by the diagnostic test. When these consequences are severe, it seems crucial to have a high confidence in the calculated data with pretest probabilities. In this situation the need for certainty may outweigh the need for interpretability. When there is low confidence in the calculated data by using pretest probabilities, it sometimes might seem better not to present normalized frequencies. However, not presenting normalized frequencies for the interpretation of accuracy parameters may complicate the judgement of clinicians, policy makers, and guideline boards whether to use the diagnostic test or not as well.

*Rotator cuff example*

The accuracy for diagnosing any rotator cuff (i.e. shoulder muscles) tears in persons for whom surgery is considered of MRI and ultrasound was assessed in a Cochrane review by Lenza at al. (2013). False negative misclassifications might lead to untreated rotator cuff tears with persisting complaints (i.e. pain, limited shoulder movement), while false positive misclassifications may ultimately lead to unnecessary surgical shoulder incisions and further diagnostic testing. The downstream consequences of misclassifications are very inconvenient at least but are not affecting more serious events such as mortality. Therefore, we might accept more uncertainty from the data calculated with pretest probabilities to aid the interpretation of accuracy parameters. In Table A1, we have calculated normalized frequencies with a representative pretest probability for rotator cuff tears in symptomatic shoulders in the Japanese general population. There may be indirectness because of the population (i.e. Japanese), because of the target condition (symptomatic shoulder versus persons suspected of rotator cuff tears whom were considered for surgery), and the prevalence range of the MRI meta-analysis did not contain the pretest probability (the prevalence ranged from 50% to 96%). When the downstream consequences are considered acceptable and not severe, such indirectness might be acceptable for the interpretation of these normalized frequencies to decide whether or not to use MRI for diagnosing rotator cuff tears in symptomatic shoulders in the Japanese population. Especially when this is the best evidence available for that particular setting.

| **Table A1 – Normalized frequencies for MRI diagnosing any rotator cuff tear in symptomatic shoulders in the general Japanese population** | | | | |
| --- | --- | --- | --- | --- |
| MRI for detecting any rotator cuff tears in a hypothetical cohort of n=1000  Summary sensitivity: 98%  Summary specificity: 79% | | | | |
| *Pretest probability* | True positive | False positive | False negative | True negative |
| 36% (symptomatic shoulders)^1^ | 353 | 134 | 7 | 506 |
|  |  |  |  |  |
| ^1^In the Japanese general population, from Yamamoto et al. (2010) | | | | |

*Cancerous pancreatic lesion example*

In the DTA review by Best et al. (2017) imaging modalities for pancreatic lesions were analyzed. Misclassifications of cancerous pancreatic lesions have severe consequences. False negative misclassifications might lead to tumor growth and early death, while false positives might lead to psychological distress, unnecessary treatment, and intervention induced complications. Different (appropriate) pretest probabilities may yield very different test performances in a somewhat wide prevalence range (see Example 3) and even small differences in false negatives might influence the decision whether or not to use a certain imaging modality in cancerous pancreatic lesions. The presented data calculated from pretest probabilities should then be interpreted at least with caution due to the severity of consequences in misclassifications. The review authors included three studies for PET diagnosing cancerous versus benign pancreatic lesions. The prevalence was 63%, 80%, and 82% in these studies and we calculated normalized frequencies with these pretest probabilities (see table A2). We also calculated normalized frequencies for the prevalence in a hospital setting (30.4%, Walter et al. [2016]). The prevalence range of the PET meta-analysis (63-82%) did not contain the 30.4% pretest probability. Therefore, the pooled accuracy estimates do not contain data about the test performance at the hospital setting pretest probability and there might possibly be some indirectness. It is now the question whether we believe that the normalized frequencies are a truthful (enough) representation of the test’s performance considering the severe downstream consequences of misclassifications.

| **Table A2 – Normalized frequencies for PET diagnosing cancerous pancreatic lesions (cancerous vs. benign)** | | | | |
| --- | --- | --- | --- | --- |
| PET for detecting pancreatic lesions (cancerous vs. benign) in a hypothetical cohort of n=1000  Summary sensitivity: 92%  Summary specificity: 65% | | | | |
| *Pretest probability* | True positive | False positive | False negative | True negative |
| 80% (median) | 736 | 70 | 64 | 130 |
| 30.4% (hospital setting)^1^ | 280 | 244 | 24 | 452 |
|  |  |  |  |  |
| ^1^Proportion of persons suspected of pancreatic cancer in a hospital setting diagnosed with pancreatic cancer, from Walter et al. 2016 | | | | |

**Example 3 – Varying normalized frequencies in high grade vesicoureteral reflux**

In the Cochrane DTA review of Shaikh et al. (2016) summary sensitivity and specificity were obtained for both ultrasound and dimercaptosuccinic acid renal scans used for detecting vesicoureteral reflux.

The authors assumed a pretest probability of 13% in a hypothetical cohort of n=1000 for high grade vesicoureteral reflux. We used the data of the ultrasound analysis for high grade vesicoureteral reflux to calculate the mean, median, interquartile range (IQR), and the range of the disease prevalence and calculated the accompanying normalized frequencies. The authors were able to include 11 studies (2498 participants) in the ultrasound analysis for high grade vesicoureteral reflux. This resulted in a summary sensitivity and specificity of 59% and 79%, respectively. From the data of the analysis (test 2 on page 115 of the DTA review), the disease prevalence of the included studies for the ultrasound analysis were calculated: 8%, 9.1%, 9.3%, 15.6%, 16.6%, 17.7%, 19.5%, 20,1%, 21,2%, 23.9%, 31%

Therefore:

Range lower limit = 8%

IQR lower limit = 9.3%

Assumed = 13%

Mean = 17.5%

Median = 17.7%

IQR upper limit = 21.2%

Range upper limit = 31%

*Calculating the normalized frequencies*

The mean, median, interquartile range, range and the assumed pretest probability were used as pretest probabilities when calculating the normalized frequencies. The sensitivity and specificity used in these calculations will remain unchanged. With this we show that normalized frequencies vary when using different pretest probabilities determined by various methods (see Table A3).

| **Table A3 – Normalized frequencies for various pretest probabilities while the sensitivity and specificity remain constant** | | | | |
| --- | --- | --- | --- | --- |
| Ultrasound for detecting high grade vesicoureteral reflux in a hypothetical cohort of n=1000  Summary sensitivity: 59%  Summary specificity: 79% | | | | |
| *Pretest probability* | True positive | False positive | False negative | True negative |
| 8% (range lower limit) | 47 | 193 | 33 | 727 |
| 9.3% (IQR lower limit) | 55 | 190 | 38 | 717 |
| 13% (assumed) | 77 | 183 | 53 | 687 |
| 17.5% (mean) | 103 | 173 | 72 | 652 |
| 17.7% (median) | 104 | 173 | 73 | 650 |
| 21.2% (IQR upper limit) | 125 | 165 | 87 | 623 |
| 31% (range upper limit) | 183 | 145 | 127 | 545 |
|  |  |  |  |  |
| IQR: interquartile range | | | | |

**References**

Best LM, Rawji V, Pereira SP, Davidson BR, Gurusamy KS. Imaging modalities for characterising focal pancreatic lesions. Cochrane Database Syst Rev. 2017 Apr 17;4:CD010213. doi: 10.1002/14651858.CD010213.pub2. Review. PubMed PMID: 28415140; PubMed Central PMCID: PMC6478242.

Lenza M, Buchbinder R, Takwoingi Y, Johnston RV, Hanchard NC, Faloppa F. Magnetic resonance imaging, magnetic resonance arthrography and ultrasonography for assessing rotator cuff tears in people with shoulder pain for whom surgery is being considered. Cochrane Database Syst Rev. 2013 Sep 24;(9):CD009020. doi: 10.1002/14651858.CD009020.pub2. Review. PubMed PMID: 24065456; PubMed Central PMCID: PMC6464715.

Shaikh N, Spingarn RB, Hum SW. Dimercaptosuccinic acid scan or ultrasound in screening for vesicoureteral reflux among children with urinary tract infections. Cochrane Database Syst Rev. 2016 Jul 5;7:CD010657. doi: 10.1002/14651858.CD010657.pub2. Review. PubMed PMID: 27378557; PubMed Central PMCID: PMC6457894

Walter FM, Mills K, Mendonça SC, Abel GA, Basu B, Carroll N, Ballard S, Lancaster J, Hamilton W, Rubin GP, Emery JD. Symptoms and patient factors associated with diagnostic intervals for pancreatic cancer (SYMPTOM pancreatic study): a prospective cohort study. Lancet Gastroenterol Hepatol. 2016 Dec;1(4):298-306. doi: 10.1016/S2468-1253(16)30079-6. Epub 2016 Oct 4. PubMed PMID: 28404200; PubMed Central PMCID: PMC6358142.

Wijedoru L, Mallett S, Parry CM. Rapid diagnostic tests for typhoid and paratyphoid (enteric) fever. Cochrane Database Syst Rev. 2017 May 26;5:CD008892. doi: 10.1002/14651858.CD008892.pub2. Review. PubMed PMID: 28545155; PubMed Central PMCID: PMC5458098.

Yamamoto A, Takagishi K, Osawa T, Yanagawa T, Nakajima D, Shitara H, Kobayashi T. Prevalence and risk factors of a rotator cuff tear in the general population. J Shoulder Elbow Surg. 2010 Jan;19(1):116-20. doi: 10.1016/j.jse.2009.04.006. PubMed PMID: 19540777.
